# Effect of a powered and a manual toothbrush in subjects susceptible to gingival recession: A 36‐month randomized controlled clinical study

**DOI:** 10.1111/idh.12834

**Published:** 2024-06-11

**Authors:** Simone Sutor, Christian Graetz, Antje Geiken, Martin Straßburger, Carolin Löwe, Bernhard Holtmann, Jonas Conrad, Sonja Sälzer, Christof E. Dörfer

**Affiliations:** ^1^ Clinic of Conservative Dentistry and Periodontology University of Kiel Kiel Germany; ^2^ Zahnarztpraxis Poststraße Hamburg Germany

**Keywords:** dental devices, home care, gingival recession/aetiology, gingival recession/prevention & control, toothbrushing

## Abstract

**Objective:**

The objective of this long‐term clinical study was to evaluate the influence of a newly developed powered toothbrush (PT) on the size and number of pre‐existing gingival recessions (GR) in comparison to a manual toothbrush (MT).

**Methods:**

This was a prospective, single‐blind, parallel‐group, randomized controlled clinical study. Participants without periodontitis, but with at least two teeth (index teeth) showing GR ≥2 mm were randomized to brush either twice daily with a MT or with a PT with a linear magnetic drive causing the round brush head to produce gentle micro vibrations along with oscillating‐rotating movements. Primary outcome parameter was the mean change of GR at the index teeth over 36 months.

**Results:**

Totally 87 out of 92 participants completed the study (MT/PT: *n* = 42/*n* = 45). At the 36‐month evaluation the mean (standard deviation) change of GR at index teeth differed significantly between MT 0.17 (0.77) and PT −0.10 (0.63) (*p* = 0.013). Furthermore, the amount of GR sites which improved ≥1 mm or remained stable during the study period did not differ between MT and PT, but the number of sites worsened ≥1 mm was significantly in favour for PT (MT 23 (25.5%) versus PT 10 (10.6%); *p* = 0.009). A binary logistic regression identified tooth type (OR = 2.991 for pre−/molar (1.096 [95% CI 1.002–8.933]; *p* = 0.050)) and manual brushing (OR = 3.341 (1.206 [95% CI 1291–8648]; *p* = 0.013)) as risk factors for recession impairment at the index teeth. There were no differences between groups for adverse events.

**Conclusion:**

In a population with pre‐existing gingival recessions and consequently a high risk of developing further recession the PT seems to be favourable with regard to further development of GR.

## 1 INTRODUCTION

It is well accepted that mechanical toothbrushing removes dental plaque and hence can reduce gingivitis parameters.^1^ In addition, evidence shows that the prevention and treatment of gingivitis helps to prevent periodontitis.^2^ Hence, daily oral hygiene is of utmost importance in preventive dentistry. For dental plaque removal, a systematic review found a high certainty for a small effect of powered toothbrushes (PT) over manual toothbrushes (MT) and with moderate certainty, a very small benefit for the use of a PT with an oscillating‐rotating over a high‐frequency sonic mode of action.^3^

Besides gingivitis and periodontitis, gingival recession (GR) is also a global public health problem effecting more than two‐thirds of the population worldwide.^4^ GR is defined by the apical displacement of the gingival margin relative to the cemento‐enamel junction (CEJ).^5^ This leads to root exposure, which is aesthetically unattractive and may eventually result in dentine hypersensitivity and root caries. GR on the buccal surface of teeth should be considered as predisposing factors for dentine hypersensitivity.^6^ The aetiology of GR is multifactorial and is an expression of a variety of anatomical, pathological and physiological factors.^5^ Several investigations found an association with aging^7^ and gender (female).^8^ Besides, toothbrushing variables, mainly excessive pressure during brushing, are discussed as potential risk factors.^9^, ^10^, ^11^ In this context, there may be some reservation about PTs as higher efficacy is linked with higher risk, although there is evidence of less pressure with PT and a systematic review refuting such an association.^12^

However, the role of toothbrushing in promoting GR is controversial. Teeth with untreated GR are at high risk for increasing the recession depth.^15^ In a previously published study the authors investigated participants with pre‐existing mid‐buccal GR and found no significant difference between PT and MT during a 12‐month observation with daily use of the products.^16^, ^17^ Therefore, it could be concluded that both brushing methods were safe with respect to gingival tissues in a population at high risk. Furthermore, PT seemed to be utilized with less force, indicated by the Bristle‐Splaying‐Index.^18^ PT, therefore, does not seem to be a risk factor for developing GR^13^, ^19^, ^20^, ^21^ or worsening of GR^16^ in long‐term use. Also, various studies have shown that PT could even provide benefits compared to manual brushing.^22^, ^23^

Recently, a modified oscillating‐rotating (O‐R) PT (Oral‐B rechargeable OP020/OR015 toothbrush, (Oral‐B iO Series 7), with an Oral‐B iO Ultimate Clean brush head, Procter & Gamble Company, Cincinnati, OH) has been introduced globally. This PT works with a novel magnetic drive causing the round brush head to perform oscillating‐rotating movements with gentle micro vibrations. Studies have shown, it has a superior effect on plaque and gingivitis compared to a MT^24^, ^25^, ^26^, ^27^, ^28^ and on plaque and gingivitis compared to a sonic toothbrush.^29^, ^30^ So far, no studies have specifically evaluated the effect of this PT in participants with pre‐existing GR. The aim of this study, therefore, was to examine the influence on pre‐existing GR following 36 months of toothbrushing with a PT compared to a reference MT on pre‐existing GR.

## 2 STUDY POPULATION AND METHODOLOGY

### 2.1 Participants and the study design

This was a single center, examiner‐blind, parallel designed randomized controlled 36‐month clinical study that compared the effects of toothbrushing with a PT to a MT (American Dental Association 46 reference flat trim brush, Chicago, IL, USA) on marginal gingival tissues under long‐term (36 months) twice‐daily use. Clinical examinations were performed at the baseline and after 12, 24 and 36 months.

All procedures were performed in accordance with the ethical standards of the institutional and national research committees (Kiel IRB: D447/20), as well as the 1964 Declaration of Helsinki and their more recent versions and were approved by the ethics committee of the medical faculty of the Christian‐Albrechts‐University of Kiel. The study was registered in the German Clinical Trials Register (DRKS00015133).

Before starting the study, verbal and written information about the study protocol were given to the participants and they provided written informed consent before enrollment. Screening, as well as the clinical investigation of the participants, was performed at the Clinic for Conservative Dentistry and Periodontology, University Hospital Schleswig‐Holstein, Campus Kiel. In order to participate in the study candidates had to demonstrate the following inclusion criteria: between 18 and 65 years of age, 16 or more scorable teeth without orthodontic appliances, crowns, bridges or implants, at least two teeth showing GR of ≥2 mm on the midbuccal surfaces (index teeth) and regularly using a MT. Candidates with poor oral hygiene (e.g. lots of soft deposits and calculus) were excluded from the study as oral hygiene is related to gingivitis and a reduction of inflammation is likely during a clinical study, which might influence GR. Further exclusion criteria were multiple carious lesions requiring treatment, non‐compliance with regard to the study design, periodontitis and/or major hard/soft tissue lesions, any physical limitations or restrictions potentially interfering with normal oral hygiene, therapy with any drug within 28 days prior to the study, self‐reported pregnancy or breast feeding, any systemic condition or significant illness, pacemakers and participation in any other oral hygiene clinical study within the last 30 days. Dental professionals and dental students were excluded from participation to avoid a bias due to professional knowledge. Smoking, on the other hand, was not an exclusion criterion for participation in this study, as there is no evidence that smokers are at an increased risk for the development of type 1 GR.^31^ Qualifying participants were consecutively included and randomly assigned using a computer software (Microsoft Access, Microsoft Corporation, Redmond, WA, USA) to one of the two brushing groups (PT or MT), after having been stratified based on the gingival phenotype, number of sites with recession ≥2 mm, gender and smoking. The product was distributed by the same study personnel (SiS, CG), which performed the randomization and was not involved in the examination.

During the first visit (Visit 1; baseline) one experienced clinical examiner (SoS), who was blinded with respect to the group assignment, assessed the probing pocket depth (PPD) at six sites per tooth (mesiobuccal, midbuccal, distobuccal, mesiolingual, midlingual and distolingual). PPD at every site was measured using a 15 mm periodontal probe (PCPUNC15, Hu‐Friedy, Chicago, IL, USA) marked at each mm and measurements were rounded off to the nearest mm. At the same time, clinical attachment level (CAL) was assessed and consequently GR calculated as the difference of CAL and PPD. GR was assessed by the same clinical examiner (SoS) at all visits with the same probe in all cases, where the gingival margin was apical to the cemento‐enamel junction (CEJ). The site was not evaluated if the CEJ was not clearly detectable due to abrasion or a restoration. In addition, the gingival phenotype was determined and categorized as thick or thin using the periodontal probe. In the thick phenotype, the periodontal probe did not show through when probing the sulcus. Whereas in the thin phenotype, the outline of the periodontal probe was visible through the gingival margin, indicating that the thickness of the gingiva in the thin phenotype was usually thinner than 1 mm.^32^, ^33^ Furthermore muco‐gingival tissues were examined regarding alteration. Plaque was assessed consecutively by the modified Turesky‐Index for further analysis (AG). The examiners had been calibrated at 10 patients, not included in the current study before hand for intra‐ and inter‐examiner reproducibility.

All participants, MT as well as PT group, were instructed orally and in writing by the two study coordinators (SiS, CG) to brush their teeth twice daily for 2 min using their habitual brushing technique with a standard sodium fluoride dentifrice (Blend‐a‐Med Classic toothpaste (NaF 1450 ppm F); Procter & Gamble Company GmbH, Schwalbach, Germany). They were instructed to brush tooth by tooth, cleaning all surfaces (outer surfaces, inner surfaces, occlusal surfaces, gingival margin) with light pressure^34^ for 30 s in each quadrant. No further instruction was given regarding to the toothbrushing technique.

Additionally, in the PT group subjects were instructed to follow manufacturer's instructions, which mainly explains functional aspects of the PT and to keep pressure in the green zone. The brushing force by PT was controlled by a sensor and the correct pressure was visible for the patient. For the ‘green zone’ a pressure of 0.8–2.5 N was defined, to little pressure (white light) <0.8 N and too high for pressure (red light) measuring >2.5 N.

As the aim of this study was to evaluate the effect of the toothbrush in GR further confounders were tried to be excluded. Hence, no further advice regarding oral hygiene aids were given. All participants were allowed to continue using rinse and interdental hygiene devices, as documented at the baseline, but were instructed not to add/change any other oral hygiene products for the study duration. Instructions were provided to all participants at the baseline and each follow‐up visit.

Every 3 months, participants were scheduled to return their used brush head or MT and tubes of dentifrice (Blend‐a‐Med Classic toothpaste (NaF 1450 ppm F); Procter & Gamble Company GmbH, Schwalbach, Germany) and to receive a supplemental kit box containing their assigned treatment products, which would last through the subsequent 3 months. At 12, 24 and 36 months, participants were appointed to the clinic for the clinical assessment and evaluation of the continuance criteria (participation in other dental product clinical studies, use of non–study toothbrushes or dentifrices, change of oral hygiene habits, use of any drugs with the potential side effect of leading to gum overgrowth, elective dentistry, including dental prophylaxis within 4 weeks prior to 12‐, 24‐ and 36‐month appointments, self‐reported pregnancy or breast feeding), an updating of the medical history and an oral examination. PPD and GR were measured at six sites (SoS) as in the baseline visit. Furthermore participant‐reported and examiner‐observed adverse events were recorded.

### 2.2 Statistical analyses

To detect a minimal statistically significant difference of 0.2 mm between the study group means with a power of 80% at an alpha of 0.05, a sample size of *n* = 40 was calculated, based on a pooled SD of 0.374 and an effect size of 0.21 mm derived from a previous study.^16^ To account for drop‐outs, a sample size of ≥50 per group was chosen.

The individual participant was the unit of analysis. The primary outcome parameter was the mean change of GR at the index teeth between the baseline and 36 months. Changes from the baseline to 36 months between the groups were assessed using the Mann–Whitney *U* test and within groups using the Wilcoxon non‐parametric test with Bonferroni correction for multiple testing.

A binary logistic regression with eight parameters was used to identify possible factors (age, sex (female vs. male), brushing group (PT vs. MT), gingival phenotype (thin vs. thick), tooth type (front vs. premolar/molar), smoking (non‐smoking vs. smoking), handedness (left vs. right)) for a recession impairment of the index teeth over 36 months.

All statistical tests were subject based, two‐sided and a level of *p* < 0.05 was considered to be statistically significant. In order to maintain a prognostic balance generated from the original random treatment allocation missing data in the primary outcome due to drop‐outs were imputed. Hereby the last observation was carried forward in order to allow for analysis on completed subject data, following the Intention to Treat (ITT) protocol.

## 3 RESULTS

**FIGURE 1:**
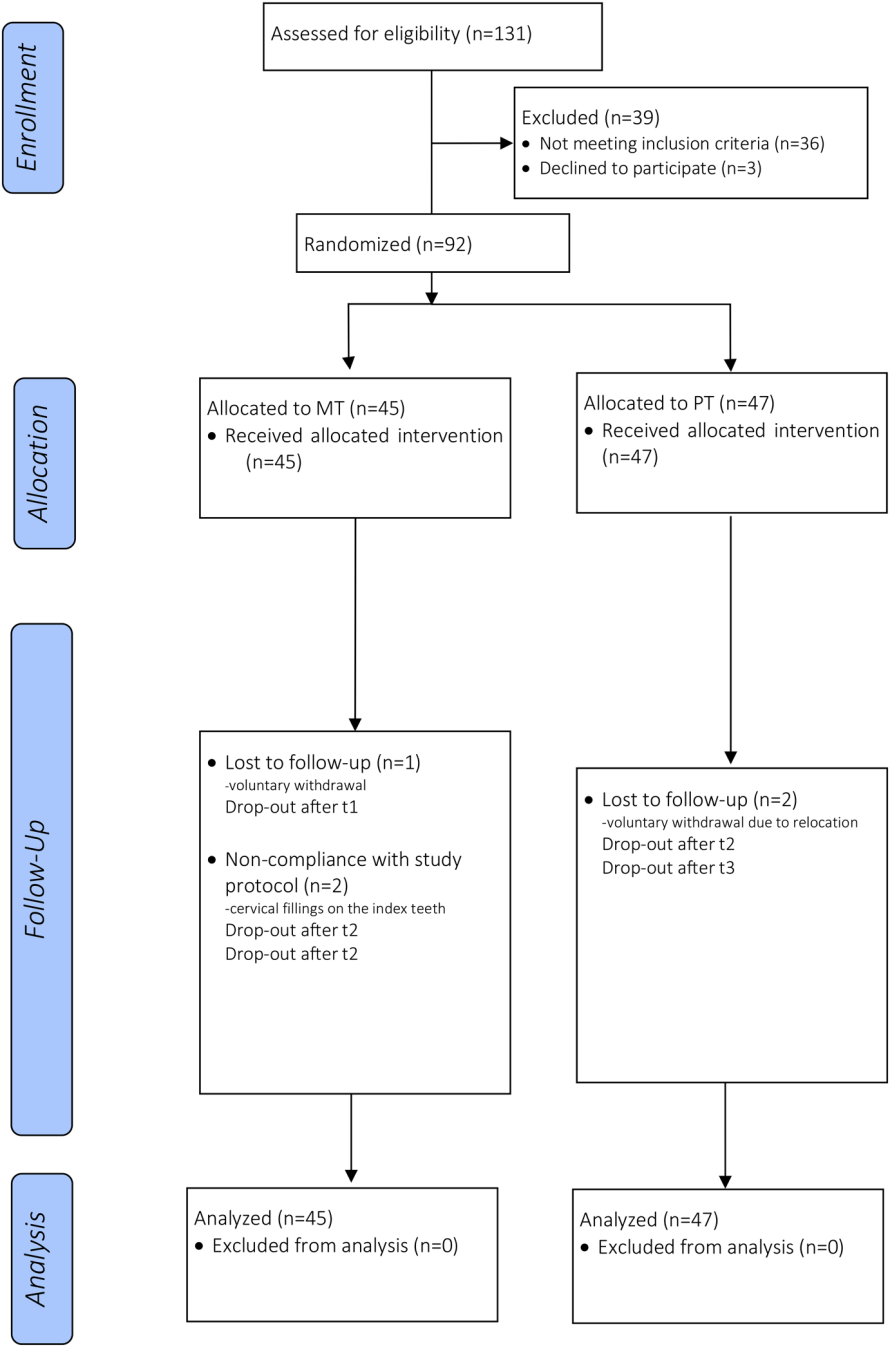
Flowchart. Participant enrollment, allocation, follow‐up and analysis (*n*: number, t1: Baseline, t2: 12‐month visit, t3: 24‐month visit, MT: ADA reference manual toothbrush, PT: powered toothbrush). The clinical data of participants who dropped out were managed according to the data imputation method.

A total of 131 participants were recruited and screened between October 8th, 2018 and February 20th, 2019. Eventually, 92 participants (MT/PT: 45/47) were randomized into the study, of which 87 (MT/PT: 42/45) completed the study (Figure 1). Three participants (1 MT/2 PT) could not return to the study center because of relocation or scheduling conflicts. Therefore, the first drop‐out occurred after 3 months in the study, the second drop‐out after the 12‐month appointment and the third drop‐out after the 24‐month appointment. Furthermore, two participants in the MT group were excluded from the study after the 12‐month appointment because of a violation of the study protocol (cervical restorations at both index teeth). In total, five participants were unavailable for the 36‐month appointment. Data for these missing participants were imputed according to the ITT protocol. Therefore, missing values from visit 12‐, 24‐ and 36‐month were replaced by the corresponding values from the previous visit (last‐observation carried forward).

### 3.1 Demographic data

The demographic details of participants enrolled for the study are summarized in Table 1. The overall mean (standard deviation) age was 44.0 (11.0) years with 67.4% females and 32.6% males. The majority of the participants in both groups were non‐smokers (MT/PT: 39 (86.7%)/42 (89.4%)). At the baseline, 31.1% of the MT and 29.8% of the PT group participants showed a thin gingival phenotype.

**TABLE 1:** Baseline demographic characteristics of participants enrolled in the study (MT: ADA reference manual toothbrush; PT: powered toothbrush).

Demographic characteristics at the baseline			
Demographic characteristics	MT (*n* = 45)	PT (*n* = 47)	Overall (*n* = 92)
Age (years)			
Mean (SD) [range]	44.8 (10.7) [25–61]	43.2 (11.3) [20–61]	44.0 (11.0) [20–61]
sex			
Female (in %)	32 (71.1%)	30 (63.8%)	62 (67.4%)
Male (in %)	13 (28.9%)	17 (36.2%)	30 (32.6%)
smoker			
Yes (in %)	6 (13.3%)	5 (10.6%)	11 (12.0%)
No (in %)	39 (86.7%)	42 (89.4%)	81 (89.0%)
Gingival phenotype			
Thin (in %)	14 (31.1%)	14 (29.8%)	28 (30.4%)
Thick (in %)	31 (68.9%)	33 (70.2%)	64 (69.6%)
Handedness			
Right‐handed (in %)	41 (91.1%)	40 (85.1%)	81 (88.0%)
Left‐handed (in %)	4 (8.9%)	7 (14.9%)	11 (12.0%)

### 3.2 Mean change at mid‐buccal sites with pre‐existing recession ≥2 mm at index teeth

The changes of Pre‐GR values of ≥2 mm were assessed at 90 sites in the MT group and at 94 sites in the PT group during the observation time of 36 months. Mean change of GR from the baseline to the final 36‐month examination at the index teeth as primary outcome as well as the 12‐ and 24‐month visits is presented in Table 2. The GR‐changes differed significantly between MT 0.17 (0.77) and PT −0.10 (0.63) (*p* = 0.013). Proportions between stable/improved vs. progressed GR at index teeth were statistically significant different in favour of PT with 67 (74.4%) for MT and 84 (89.3%) PT (*p* = 0.039).

**TABLE 2:** Distribution of the number of GR and changes of the values of GR at the baseline (t1), 12 months (t2), 24 months (t3) and 36 months (t4) at index teeth (MT/PT: *n* = 90/*n* = 94) (MT: ADA reference manual toothbrush, PT: powered toothbrush).

Group	Baseline (t1)	12 months (t2)	24 months (t3)	36 month (t4)	t1–t2	t1–t3	t1–t4
Mean (SD) GR (mm) of index teeth (GR ≥2 mm)							
					**GR‐change**	**GR‐change**	**GR‐change**
MT	2.49 (0.62)	2.62 (0.71)	2.64 (0.75)	2.66 (0.77)	0.13 (0.66)	0.16 (0.72)	0.17 (0.77)
PT	2.61 (0.83)	2.57 (0.78)	2.43 (0.86)	2.50 (0.68)	−0.02 (0.61)	−0.17 (0.70)	−0.10 (0.63)
Between group analysis MT versus PT *p*‐value*	*p* = 0.498	*p* = 0.511	*p* = 0.062	*p* = 0.150	*p* = 0.110	***p* = 0.001**	***p* = 0.013**
Within group analysis of GR‐changes *p*‐value**	**MT**				1.000	1.000	1.000
	**PT**				1.000	0.898	1.000
*N* of (%) index teeth (GR ≥2 mm) with improved GR							
MT	90 (100%)	90 (100%)	89 (98.9%)	90 (100%)	12 (13.3%)	9 (10%)	12 (13.3%)
PT	94 (100%)	94 (100%)	90 (95.7%)	94 (100%)	16 (17.1%)	21 (22.3%)	18 (19.1%)
Between group analysis MT versus PT *p*‐value*	1.000	1.000	1.000	1.000	*p* = 0.487	***p* = 0.024**	*p* = 0.287
Within group analysis of N of improvement *p*‐value**	MT				1.000	1.000	1.000
	PT				1.000	1.000	1.000
*N* of (%) index teeth (GR ≥2 mm) with stable GR							
MT					56 (62.2%)	60 (66.7%)	55 (61.1%)
PT					64 (68.1%)	65 (69.1%)	66 (70.2%)
Between group analysis MT versus PT *p*‐value*					*p* = 0.405	*p* = 0.719	*p* = 0.195
Within group analysis of N of stable *p*‐value**	**MT**				1.000	1.000	1.000
	**PT**				1.000	1.000	1.000
*N* of (%) index teeth (GR ≥2 mm) with worsened GR							
MT					22 (24.4%)	21 (23.3%)	23 (25.5)
PT					14 (14.9%)	8 (8.5%)	10 (10.6%)
Between group analysis MT versus PT *p*‐value*					*p* = 0.104	***p* = 0.006**	***p* = 0.009**
Within group analysis of N of worsened *p*‐value**	**MT**				1.000	1.000	1.000
	**PT**				1.000	1.000	1.000

*Note*: The bold values indicate the significant *p*‐values.

* Mann–Whitney *U* test.

** Wilcoxon test with Bonferroni correction for multiple testing.

In detail, a decrease in these GR of at least 1 mm was observed after 36‐months in 12 sites (13.3%) in the MT group versus 18 sites (19.1%) in the PT‐group (*p* = 0.287). The frequency of GR with an increase of GR ≥1 mm (MT 23 (25.5%) versus PT 10 (10.6%); *p* = 0.009) was found to be statistically significant, in favour of PT.

**FIGURE 2:**
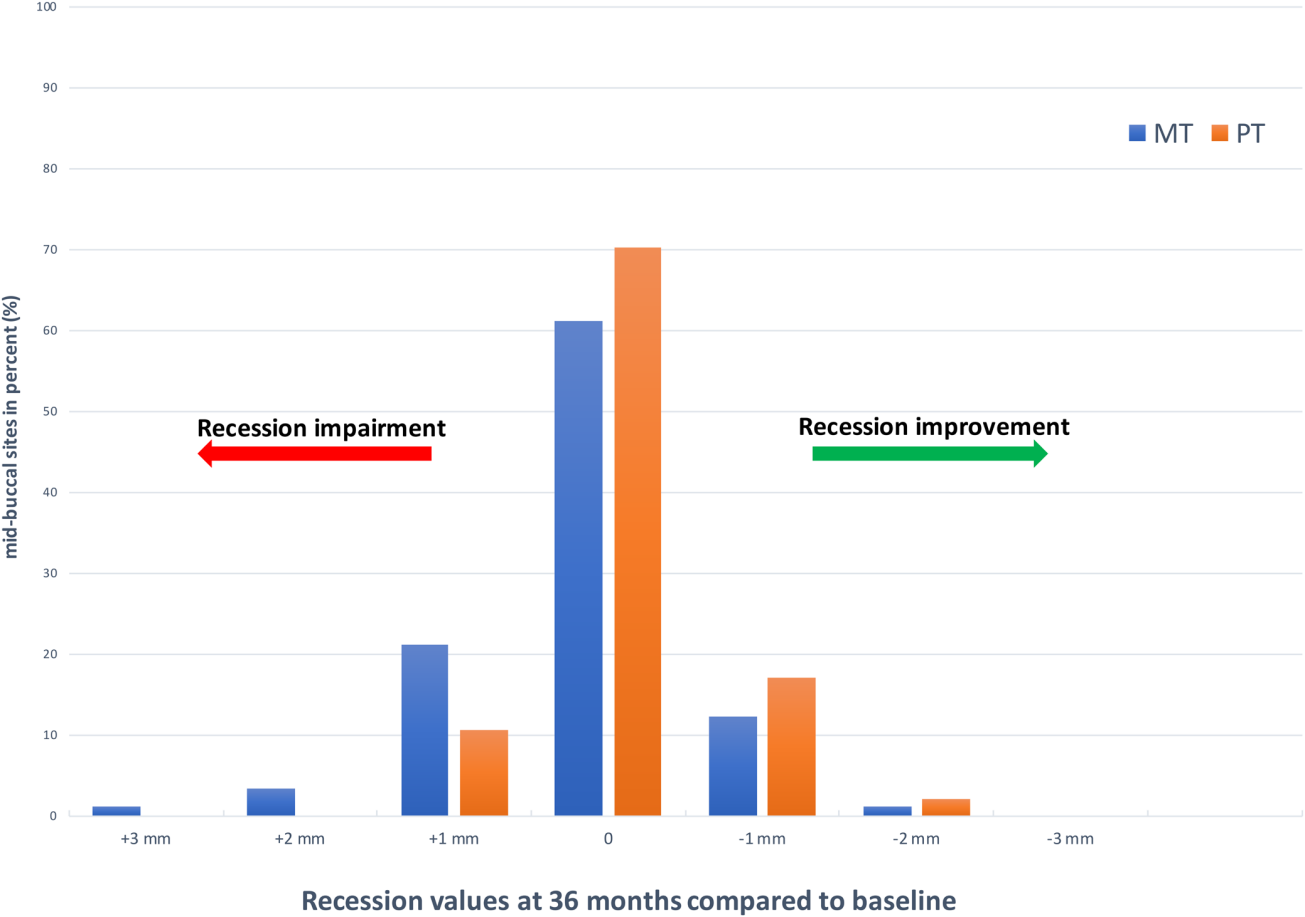
Mid‐buccal sites at index teeth (MT: *n* = 90; PT: *n* = 94) with pre‐existing GR sites ≥2 mm demonstrating an improvement or impairment of ≥1 mm from the baseline to final examination at 36 months (MT: ADA reference manual toothbrush, PT: powered toothbrush).

A decrease in GR of at least 2 mm was observed in 1 (1.1%) site in the MT group and in 2 (2.1%) sites in the PT group (*p* = 0.587), whereas in PT no site had an increase of at least 2 mm (MT: 4 (4.4%); *p* = 0.039). Similar we found for an increase of at least 3 mm (PT vs. MT: 0 (0%) vs. 1 (1.1%); *p* < 0.001), whereas neither in MT nor in PT a decrease of at least 3 mm was measured. All results according to the number of index teeth with in‐ and decreasing recession values were illustrated in Figure 2.

A multivariate logistic regression analysis identified two risk factors for recession impairment at the index teeth; (1) MT (*B* = 1.206; *p* = 0.013) and pre−/molars (*B* = 1.096; *p* = 0.050) are at higher risk for impairing GR compared to PT and incisors (Table 3).

**TABLE 3:** Results of binary logistic regression analysis for the significant predictors for an impairment (≥1 mm) of the GR of the index teeth.

Variables	*B*	SE	OR	95% CI		*p*‐value
				Lower limit	Upper limit	
Age at the baseline (years)	0.018	0.022	1.018	0.975	1.063	0.412
Gender (reference: female)	0.388	0.486	1.475	0.569	3.819	0.424
Pre‐GR at the baseline (mm)	−0.738	0.425	0.478	0.208	1.099	0.082
Brushing group (reference: power brushing)	1.206	0.485	3.341	1291	8648	0.013*
Tooth type (reference: front teeth)^a^	1.096	0.558	2.991	1.002	8.933	0.050*
Gingival phenotype (reference: thick)	0.246	0.495	1.279	0.485	3.373	0.619
Smoking (reference: non‐smoking)	0.240	0.656	1.271	0.351	4.598	0.715
Handedness (reference: right handed)	−0.161	0.826	0.851	0.168	4.299	0.845
Constant	−1.866	2.041	0.155			0.361

Abbreviations: *B*, Regression coefficient; SE, standard error; OR, odds ratio.

^a^ Tooth type (front teeth versus posterior teeth (pre‐ and molars)).

* Significant at *p* ≤ 0.05.

### 3.3 Overall data

The mean baseline PPD was shallow with 1.97 (0.18) mm and did not change significantly during the 36‐month observation time (2.00 (0.21) mm at 12 months, 2.02 (0.19) mm, 24 months, or 2.04 (0.19) mm 36 months (*p* > 0.05)). Within groups, we found for MT a significant increase in PPD between the baseline and 36 months (*p* = 0.015), but no statistically significant differences in the PPD mean values were observed at each visit between MT and PT groups (*p* > 0.05). For bleeding on probing (BOP per subject in percent), no significant differences could be detected neither at the baseline (MT/PT: 19.63 (12.93)%/17.63 (17.04)%) nor at any follow‐up visit, as shown in Table 4. Also, we could not detect any statistically significant difference between groups for mean CAL values at each visit, but we found a statistically significant difference between the baseline and 36‐months in the PT group (*p* = 0.050) with reduced GR. No differences between groups were found with respect to adverse events.

**TABLE 4:** Pocket probing depth (PPD), clinical attachment level (CAL) values and bleeding on probing (BOP) at the baseline (t1), 12 months (t2), 24 months (t3) and 36 months (t4) of all teeth (MT: ADA reference manual toothbrush, PT: powered toothbrush).

Group	Baseline (t1) (mean (SD))	12 months (t2) (mean (SD))	24 months (t3) (mean (SD))	36 months (t4) (mean (SD))
PPD in mm				
MT	1.96 (0.18)	1.98 (0.15)	2.03 (0.19)	2.04 (0.20)
PT	1.98 (0.19)	2.01 (0.24)	2.02 (0.18)	2.03 (0.18)
Between group analysis MT versus PT *p*‐value*	*p* = 0.735	*p* = 0.955	*p* = 0.797	*p* = 0.609
CAL in mm				
MT	2.11 (0.18)	2.16 (0.19)	2.20 (0.21)	2.21 (0.21)
PT	2.14 (0.19)	2.18 (0.24)	2.18 (0.20)	2.18 (0.18)
Between group analysis MT versus PT *p*‐value*	*p* = 0.667	*p* = 0.794	*p* = 0.773	*p* = 0.794
BOP in %				
MT	19.63 (12.92)	19.69 (13.58)	21.20 (14.11)	21.88 (14.73)
PT	17.63 (17.04)	19.11 (19.44)	18.65 (12.76)	17.40 (14.45)
Between group analysis MT versus PT *p*‐value*	*p* = 0.195	*p* = 0.283	*p* = 0.373	*p* = 0.092

* Mann–Whitney *U* test.

## 4 DISCUSSION

In this study, an O‐R PT with a newly developed linear magnetic drive causing the round brush head to produce gentle micro vibrations along with O‐R movements, was used. So far, no study has been published evaluating the effect of the investigated toothbrush on GRs. As it might be challenging for a regular PT user to adhere for 3 years to a MT and hence, to avoid violation of the study protocol, regular PT users were excluded from enrollment for the study. This resulted in a low dropout rate of only five participants over the 36‐month study period due to other reasons.

The primary aim of this study was to compare changes in Pre‐GR ≥2 mm after using a PT over a period of 36 months compared to the use of a MT at the index teeth. Both, the group analysis, as well as the secondary multivariate regression analyses showed a statistically significant benefit of PT over MT with respect to GR progression in such subjects. The mean changes of clinical recessions during the observation period of 36 months in this study was clinically small although statistically significant. For the individual patient a change of clinical recession >1 mm is however of relevance and such an impairment was only observed for MT. The present findings are in agreement with previous results on oscillating rotating toothbrushes as presented in a systematic review^12^ and several long‐term studies on various power toothbrush types demonstrating that the changes in recession are not different from a reference‐MT.^13^, ^16^, ^19^, ^20^, ^21^, ^35^

In agreement with the results of the present study a slight improvement in recession had been reported previously.^16^, ^17^, ^19^, ^20^ Hence, we concluded, that the general reduction of the Pre‐GR in PT was most likely due to improved toothbrushing over 3 years of use. In contrast, Ganss et al. showed in a randomized video observation study of 95 recorded subjects that with both MT and PT mostly horizontal and circling movements were observed, whereas 50.5% of the subjects spent only <10% of the brushing duration with the recommended passive brushing for PT. It was also shown that regardless to the type of toothbrush oral areas were reached significantly less than vestibular areas (*p* ≤ 0.001, 44.2% MT, 58.9% PT). The authors concluded that intra‐individual motion patterns were similar with both MT and PT and most subjects did not adapt brushing performance to the type of toothbrush and that probably proper instruction using a PT is necessary.^36^ Hence the brushing force by PTs in this trial is controlled by a sensor and the correct pressure is visible for the patient, it is most likely lower as compared to MTs.^37^ PTs with remote displays or apps^38^, ^39^ as well as feedback software to self‐monitor the brushing technique^40^ might be useful tools to create similar effects on brushing behaviour and to help establish correct brushing techniques avoiding GR. A recently published study by Thurnay et al. using app data from an interactive oscillating‐rotating toothbrush strongly supports these assumptions. The study showed that the use of app technology in combination with a PT leads to an improved brushing behaviour and helps reaching and maintaining gingival health, particularly when used with live feedback.^37^

However, the underlying mechanisms for such a reduction in recession remain unclear. An increasing patient awareness about GR susceptibility and modifiable conditions is said to be an indispensable first step for prevention.^41^ It is still not known which is the best brushing technique for patients with GRs. However, it is known, that the horizontal scrubbing technique is associated with GRs.^42^ Although participants did not receive a hands‐on brushing technique instruction and were not informed on the aetiology of GRs during this study, they became aware that they have GRs and that the effect of brushing was being monitored. This might have led to an improved brushing technique by the ‘Hawthorne effect’.^43^, ^44^ As discussed by our group in a previous investigation^16^ and recommended by McCracken et al.^21^ future studies should be sufficiently powered to detect smaller effect sizes and brushing behaviours should be monitored by using the smart features that come with PTs.

Studies indicate that sites with Pre‐GR are more likely to develop further recession.^14^, ^45^ A systematic analysis of the long‐term results of 1647 untreated teeth with GR at the baseline showed that 78.1% experienced an increase in GR depth during the follow‐up period, while the remaining experienced a decrease or no change.^15^ Chambrone, Tatakis^15^ pointed out that untreated GR defects in individuals with good oral hygiene have a high probability of progressing during long‐term follow‐up. Therefore, the current study was performed in order to investigate the influence of the tested toothbrushes on teeth at high risk of GR in long‐term, hence change of pre‐GR was the primary analysis in our study. In order to mitigate/reduce potential errors or bias in estimating the position of the cemento‐enamel junction—and therefore improve the predictability of the investigation results—two index teeth with a clearly defined cemento–enamel junction were chosen. Hence, while with the study design were more likely to find an effect of toothbrushing on GRs, this effect might be overestimated for the general population without recession type 1 defect.

Therefore, it may be seen as a limitation that we focused on sites with pre‐existing recession, e.g. buccally positioned roots in combination with a thin gingival phenotype, which are attributed to a high risk of recession worsening. On the other hand, this may also be seen as strength as those areas challenge the used devices and brushes to keep such defects stable and to prevent them from further progression. Another limitation of the study could be seen in the lack of instructions on a particular toothbrushing technique by dental professionals in either group. However, the subjects were deliberately not instructed in toothbrushing technique to maintain, as far as possible, the same brushing habits as before the start of the study. Hence, according to the study protocol, only participants, who had mainly brushed with a MT before the start of the study, were included. There was no professional instruction and demonstration on the use of the PT simulating a first‐time retail purchase. Therefore, the instructions focused only on duration, systematics and pressure and it is likely that only a minority of participants were in compliance with all of these criteria.^34^

As no differences in adverse events were seen between PT and MT, PT could be stated to be safe in such pathologies. Furthermore, the clinical investigator in our study was not blinded to the time point of the study, which might have influenced the assessment and resulted in regression to the mean.^46^ But as previous studies of our group have shown,^16^ this limitation is rather negligible, as Sälzer et al. 2017 showed in a 12‐month RCT using stone‐cast replica assessments that this possible limitation had no influence on the results of the study.^16^ Correspondingly, sites with Pre‐GR tended to be less extreme at the end of the study.^16^

## 5 CONCLUSION

In this study, we chose participants with pre‐existing GR as they have already shown of being at high risk for recession formation and, therefore, are likely to develop further recession. Our results show that in such a high risk population PT seems to be favourable with regard to further development of GR. It may be extrapolated that PT be also protective in patient with a lower risk.

## 6 CLINICAL RELEVANCE

### 6.1 Scientific rationale for the study

Evidence on the relationship between different types of toothbrushes and the development of GR is limited. This evaluated the long‐term influence of a powered and a MT on the development of GRs.

### 6.2 Principal findings

In comparison to a MT, twice daily use of a PT over 36 months resulted in statistically significant but small decrease of pre‐existing GR.

### 6.3 Practical implications

When recommending a toothbrush, a PT, preferably with pressure control and feedback software to self‐monitor brushing technique, should be preferred over a MT. Further research is indicated.

## AUTHOR CONTRIBUTIONS

Simone Sutor, Christian Graetz, Prof. Dr., Sonja Sälzer, PhD Dr.: conceived the ideas, collected the data, analysed the data and led the writing of the manuscript. At the time of the project, the author Simone Sutor was a PhD student. Antje Geiken, Dr., Martin Straßburger, Carolin Löwe, Bernhard Holtmann, Jonas Conrad, Dr.: collected the data and helped with writing of the manuscript. Christof E. Dörfer, Prof. Dr.: contributed to conception and design, analysis and interpretation and critically revised the manuscript.

## FUNDING INFORMATION

This study was financially supported by The Procter & Gamble Company, which provided dentifrices and toothbrushes as well.

## CONFLICT OF INTEREST STATEMENT

Prof. Dr. Dörfer and Dr. Sälzer report personal fees from Procter & Gamble, outside the submitted work. The other authors have stated explicitly that there are no conflicts of interest in connection with this article.

## Data Availability

The data that support the findings of this study are available from the corresponding author, Sonja Sälzer, PhD. Dr., upon reasonable request.
